# *OsVIT2* Mutation Increases Fe and Zn of Grain Without Compromising the Growth in Paddy Field

**DOI:** 10.3389/fpls.2022.868661

**Published:** 2022-06-22

**Authors:** Prashant Kandwal, Toru Fujiwara, Takehiro Kamiya

**Affiliations:** Laboratory of Plant Nutrition and Fertilizers, Department of Applied Biological Chemistry, Graduate School of Agriculture and Life Sciences, The University of Tokyo, Tokyo, Japan

**Keywords:** hidden hunger, biofortification, Fe, Zn, OsVIT2

## Abstract

Nearly 2 billion people who reside in developing countries are suffering from nutrient deficiency, also known as hidden hunger. A hidden hunger includes iron (Fe) and zinc (Zn) deficiency. One of the most efficient solutions to hidden hunger is the biofortification of crops through breeding. In this study, we characterized the mutant 1095_k, which has high grain Fe (~1.4-fold) and Zn (~1.2-fold) concentration compared with wild-type plants for a 5-year field trial. The yield components of 1095_k are similar to wild-type plants in a paddy field. In addition, 1095_k has a non-sense mutation in *OsVIT2*, a vacuolar localized Fe transporter. F2 crosses between 1095_k and wild type having the mutation showing higher grain Fe and Zn concentration. In contrast, plants without the mutation showed similar element concentrations as the wild type. These results suggest that *OsVIT2* would be responsible for high Fe and Zn of grain and the 1095_k would be a useful breeding material for the biofortification of Fe and Zn.

## Introduction

Micronutrients are the inorganic nutrients that are required by a living organism in a small amount to sustain its life. The deficiency of micronutrients is also known as hidden hunger which is becoming a global burden by affecting more than two billion people, nearly one-third of the world population (Bailey et al., 2015; Harding et al., 2018). Among the micronutrients, the deficiency of iron (Fe) and zinc (Zn) is one of the leading public health concerns (Harding et al., 2018). Anemia is the well-known symptom of Fe deficiency in which hemoglobin count gets reduced, and the body has difficulty meeting the oxygen demand (Owais et al., 2021). The prevalence of anemia is higher in South Asia, Southeast Asia, and sub-Saharan Africa, where women of the reproductive age group suffer more because of the menstrual cycle and pregnancy (Sunuwar et al., 2020). In addition to Fe deficiency, the deficiency of Zn results in varying adverse effects, such as stunting, impaired reproduction, immune disorders, and mortality rate of diseases (e.g., diarrhea, malaria, and pneumonia) (Berhe et al., 2019; Palanog et al., 2019). Within Africa, Zn deficiency results in 14.4% of deaths due to diarrhea, 10.4% due to malaria, and 6.7% of deaths due to pneumonia (Berhe et al., 2019).

The deficiency of micronutrients, Fe and Zn, can be accounted for various reasons, such as poor dietary intake, malabsorption, food insecurity, and non-affordability of the nutrient-rich diet (Khush et al., 2012; Gupta et al., 2020). Rice is a staple food and rich in energy but not in micronutrients. In a developing country, where rice is a staple food, people consume rice and intake energy from it but consume fewer animal products and vegetables enriched in nutrients (Van Der Straeten et al., 2020). To overcome the deficiency, biofortification of rice has been carried out using conventional breeding, an agronomic approach, and genetic modifications. The practice of crossing beneficial trait lines over several generations and selection of beneficial lines by their phenotype is the basics of conventional breeding (Kumar et al., 2019). In the agronomic approach, direct application of micronutrients either into the soil or foliar application on the plants is performed (Cakmak and Kutman, 2018). However, both conventional breeding and agronomic approaches are slow to process the release of a new crop variety (Shi et al., 2013; Ahmar et al., 2020). To overcome the limitations, the development of genetically modified organisms (GMOs) has been adopted. The transgenic approach allows the increase of micronutrients and can further introduce novel traits into plants (Christou and Twyman, 2004). Many efforts have been made to biofortify the Fe and Zn in rice, for instance, an increase in Fe content in rice by expressing the Nicotianamine aminotransferase (*NAAT*) gene (Takahashi et al., 2001), overexpression of *Oryza sativa* Nicotianamine synthase (*OsNAS2*) and *OsNAS3* (Lee et al., 2009, 2011), the introduction of soybean ferritin gene (*SoyferH1*) into rice (Goto et al., 1999), an increase of Fe and Zn in rice by expressing barley genes involved in phytosiderophore synthesis (Masuda et al., 2008). Using T-DNA insertion lines or CRISPR/Cas9 lines of rice tonoplast-localized transporters named Vacuolar Iron Transporter, OsVIT1, and OsVIT2, results in the increase of rice grain Fe and Zn content (Zhang et al., 2012; Bashir et al., 2013; Che et al., 2021). All these lines are GMOs, which are facing mass rejection, therefore limiting their availability in the market (Dipti et al., 2012). In addition, in most of the reports, yield in an actual paddy field remains to be tested (Goto et al., 1999; Lee et al., 2009, 2011; Zhang et al., 2012; Bashir et al., 2013; Che et al., 2021).

In recent years, different approaches, such as chemical-induced mutations, gamma radiation, and fast neutron-mediated mutagenesis have been adopted to improve the rice character, such as early flowering, tolerance to salinity, and drought (Sevanthi et al., 2018; Kumawat et al., 2019; Abdelnour-Esquivel et al., 2020). Once we identify the gene responsible for a phenotype, mutants are applicable for marker-assisted breeding (Karunarathna et al., 2021). Furthermore, mutants are not GMOs and are easily accepted by the market (Grover et al., 2020).

In the current study, we characterized a rice ethyl methanesulfonate (EMS)-mutagenized rice, named 1095_k, which showed a high grain Fe and Zn phenotype in paddy fields. It was found that mutant 1095_k has a nonsense mutation in *OsVIT2*. Furthermore, the mutation of *OsVIT2* did not affect yield in the fields. These results indicate 1095_k is a candidate for breeding high Fe and Zn content rice and *OsVIT2* mutation is used for marker-assisted breeding.

## Materials and Methods

### Plant Material and Growth Conditions

Field experiments were performed in the paddy field of the Institute of Sustainable Agroecosystem Services, the University of Tokyo (hereafter referred to as Tokyo field) (35°44′19.5″N 139°32′31.6″E) and Experimental Farm Station of Graduate School of Life Sciences, Tohoku University (hereafter referred to as Miyagi field) (38°15′24.4″N 140°51′29.6″E) during rice cultivation season. For the F2 population (225 lines) between WT and 1095_k grown in the Yayoi campus of The University of Tokyo (hereafter referred to as Yayoi field) (35°43′01.2″N 139°45′45.4″E).

### Determination of Fe and Zn Concentration

Five seeds for each genotype were selected to determine the Fe and Zn concentrations after removing the husk. After the measurement of dry weight, grains were applied to HNO_3_ digestion in PYREX® tubes (Iwaki) as described. Two milliliters of HNO_3_ (Wako) were applied to the tubes and heated for an initial 1 h at 70°C and then 120°C. After complete evaporation of HNO_3_, 1 ml of HNO_3_ was added, and the same temperature setting was applied. After complete evaporation of HNO_3_, 1 ml of hydrogen peroxide (H_2_O_2_) was applied to the samples until they evaporated. Finally, the samples were dissolved in a 0.08 N HNO_3_ solution and used for element concentration determination using the Inductive Coupled Plasma Mass Spectrometry (ICP-MS, Agilent 7800; Agilent Technologies).

For the polishing of rice, 15 seeds from WT and 1095_k were polished using a Pearlest grain polisher (Kett) for 1 cycle of 40 s.

### Agronomic Trait Measurement

For agronomic trait measurements, WT and 1095_k were grown in the Tokyo field. The harvesting of the WT and 1095_k plant was performed 3 cm above the ground level. The plant height was measured from the tip of the panicle to the harvested end of the plant using a regular scale. The panicle from each tiller has been removed right from the panicle base and the length was measured. The number of ripe panicles was counted.

### DNA Isolation, Sequencing, Real-Time PCR (RT-PCR), and Quantitative Real-Time PCR (qRT-PCR)

For DNA isolation, plant leaves of 15-days seedlings were used. The leaves were crushed using Zirconia beads using 300 μl of TPS buffer (100 mM Tris-HCl pH 6.8, 1 mM EDTA pH 8.0, 1.2 M KCl) followed by centrifugation at 10,000 × *g* for 13 min at 4°C. A 200 μl of the sample was transferred into the fresh tubes, and an equal volume of isopropylalchol was added. The solution was mixed and centrifuged at 10,000 × *g* for 30 min at 4°C. The supernatant was removed, and 70% ethanol was added, followed by centrifugation at 10,000 × *g* for 15 min. Again, the supernatant was removed, and the tubes were dried for not more than 10 min. The pellet was dissolved with 100 μl of TE buffer (10 mM Tris-HCl pH 8.0, 0.1 mM EDTA pH 8.0).

To determine the genomic sequence of *OsVIT1* (LOC_Os04g38940), the region containing the promoter (2,078 bp) and downstream sequence (1,002 bp), was amplified by using a forward primer 5′-CGTGCCGGAGAGAAGAACTA-3′ and a reverse primer 5′-GAATGGTTTCTGCAAGCTGGG-3′. The sequence of the *OsVIT1* genome was determined by the primers in Table S1 (Primer ID 3–12) using Sanger sequencing. For the determination of the *OsVIT2* (LOC_Os09g23300) genomic sequence, the region containing the promoter (2,035 bp) and downstream sequence (2,047 bp) was amplified by using a forward primer 5′-GACCGGTTAATTTCCCAACCG-3′ and a reverse primer 5′-AACCGACGGCACCGATCTAC-3′. The genome sequence is determined by the primers in Table S1 (Primer ID 15–23) using Sanger sequencing.

The genotype of the F2 generation was determined by derived cleaved amplified polymorphic sequences (dCAPS) marker. The DNA was isolated from the F3 bulk (6 seeds). The *OsVIT2* genomic fragment was amplified with 5′-CCGCGGAGATCGCGGACATACTGTCGCACTA-3′ and 5′-AATTAGTTTTTTCCCCTTACTTCATC-3′ (Primer ID 30 and 31 in Table S1), which introduce a DdeI site. The DdeI digested PCR product was applied to 3% agarose gel.

For real-time quantification, plants were germinated for 1 week in tap water followed by another 1 week in Kimura B solution (Uraguchi et al., 2009). RNA extraction was performed by the NucloSpin RNA plant (TaKaRa Bio). The relative quantification of *OsVIT2* was performed using a forward primer 5′-GGTATCTGGCGGCGAAGAG-3′ and a reverse primer 5′-CGACAGTATGTCCGCGATCT-3′ using the Thermal Cycler Dice Real-Time System III (TaKaRa Bio). Relative expression levels were calculated against the reference gene, *Ubiquitin* (*OsUBQ10*), amplified with a forward primer 5′-AACCAGCTGAGGCCCAAGA-3′ and a reverse primer 5′-ACGATTGATTTAACCAGTCCATGA-3′.

## Results

### Mutant 1095_k Showed High Grain Fe and Zn in Different Field Conditions

**Figure 1:**
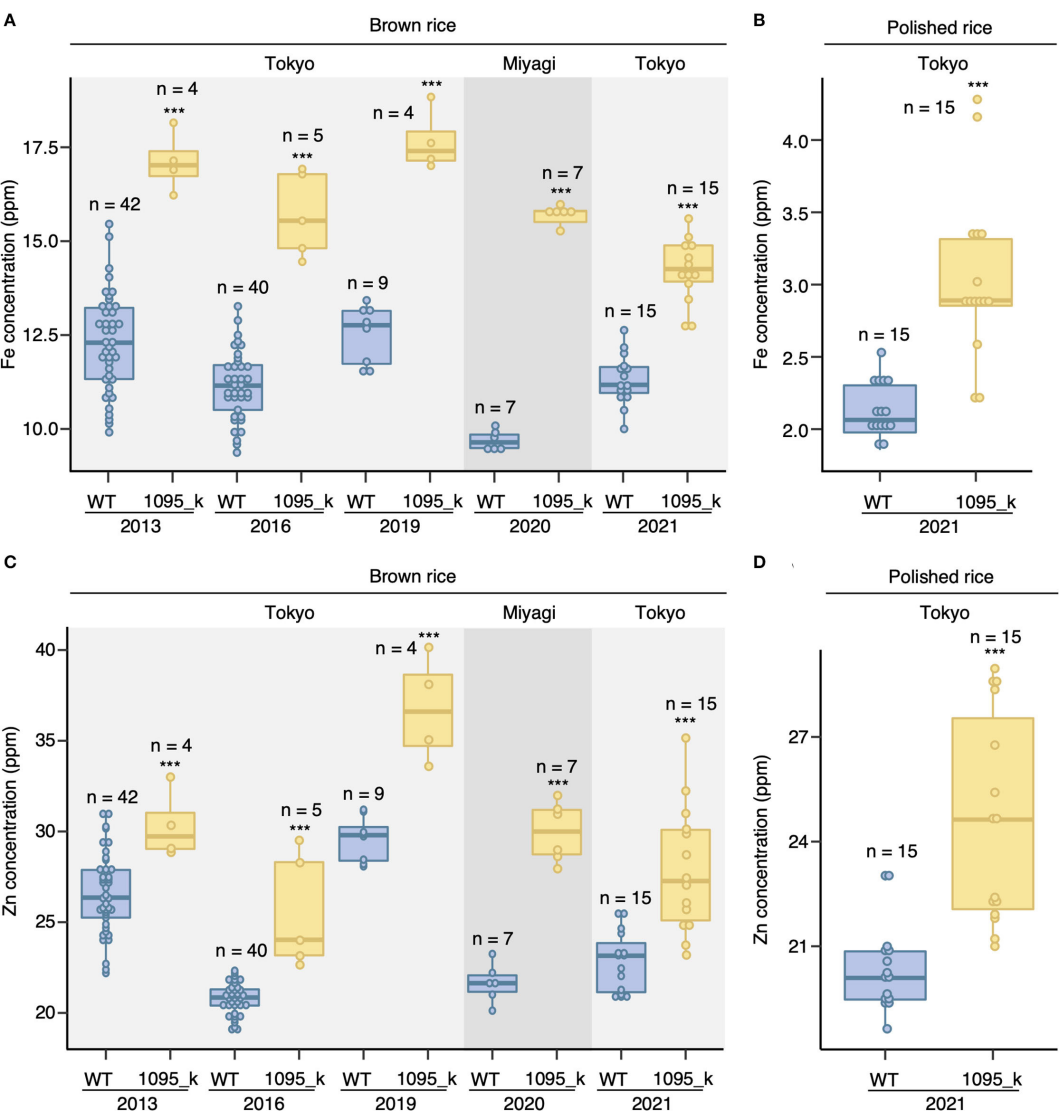
Brown rice Fe and Zn concentration. **(A)** Fe concentration in brown rice, **(B)** Fe concentration in polished rice, **(C)** Zn concentration in brown rice, and **(D)** Zn concentration in polished rice of wild type (WT) Hitomebore and mutant (1095_k). Plants were cultivated in 5 independent years on the paddy field of the Institute of Sustainable Agro-ecosystem Services, The University of Tokyo, in Tokyo, and on the field of Tohoku University in Miyagi. The number of replicates is shown in figure. Significant differences were calculated using Student's *t*-test. *** *p* < 0.001.

The EMS mutant 1095_k was isolated from the EMS mutant of *Oryza sativa* cv. Hitomebore population through ionome screening (Tanaka et al., 2016). The consistency of the mutant (1095_k) phenotype was assessed by growing both Hitomebore [wild type (WT)] and 1095_k in the paddy field condition. Both WT and 1095_k lines were grown in four independent years in the Tokyo field. After harvesting, ionome analysis was done using ICP-MS. It was found that the mutant 1095_k has a 1.4-fold higher grain Fe concentration compared with the WT (Figure 1A: Tokyo). To observe the consistency in the phenotype in a different field, WT and 1095_k were grown in the Miyagi field (Figure 1A: Miyagi). In addition, 1095_k showed a 1.5-fold higher grain Fe concentration compared with WT. Besides Fe, 1095_k has a 1.2-fold higher grain Zn concentration than WT (Figure 1B: Tokyo). In the Miyagi field, 1095_k showed a 1.5-fold higher grain Zn concentration compared with WT (Figure 1B: Miyagi). High Zn phenotype was observed in 2013, 2016, 2019, and 2021 in the Tokyo field. These results indicate that 1095_k has high Fe and Zn concentration in brown rice independent of field condition.

Hence, as commercial rice is available in the polished form, we determine the elemental concentrations of polished rice harvested in the year 2021. Polishing of brown rice resulted in ~11.37% decline in the weight of WT grains and ~12.01% decline in 1095_k grains. The polished 1095_k grains showed a 1.4-fold increase in Fe and a 1.2-fold increase in Zn concentration compared with WT (Figures 1B,D).

Apart from Fe and Zn concentration, little difference in toxic elements, such as Cd and As, was observed between WT and 1095_k in both brown rice and polished rice (Figure S1).

### Yield Components Are Similar Between WT and 1095_k in the Paddy Field

**Figure 2:**
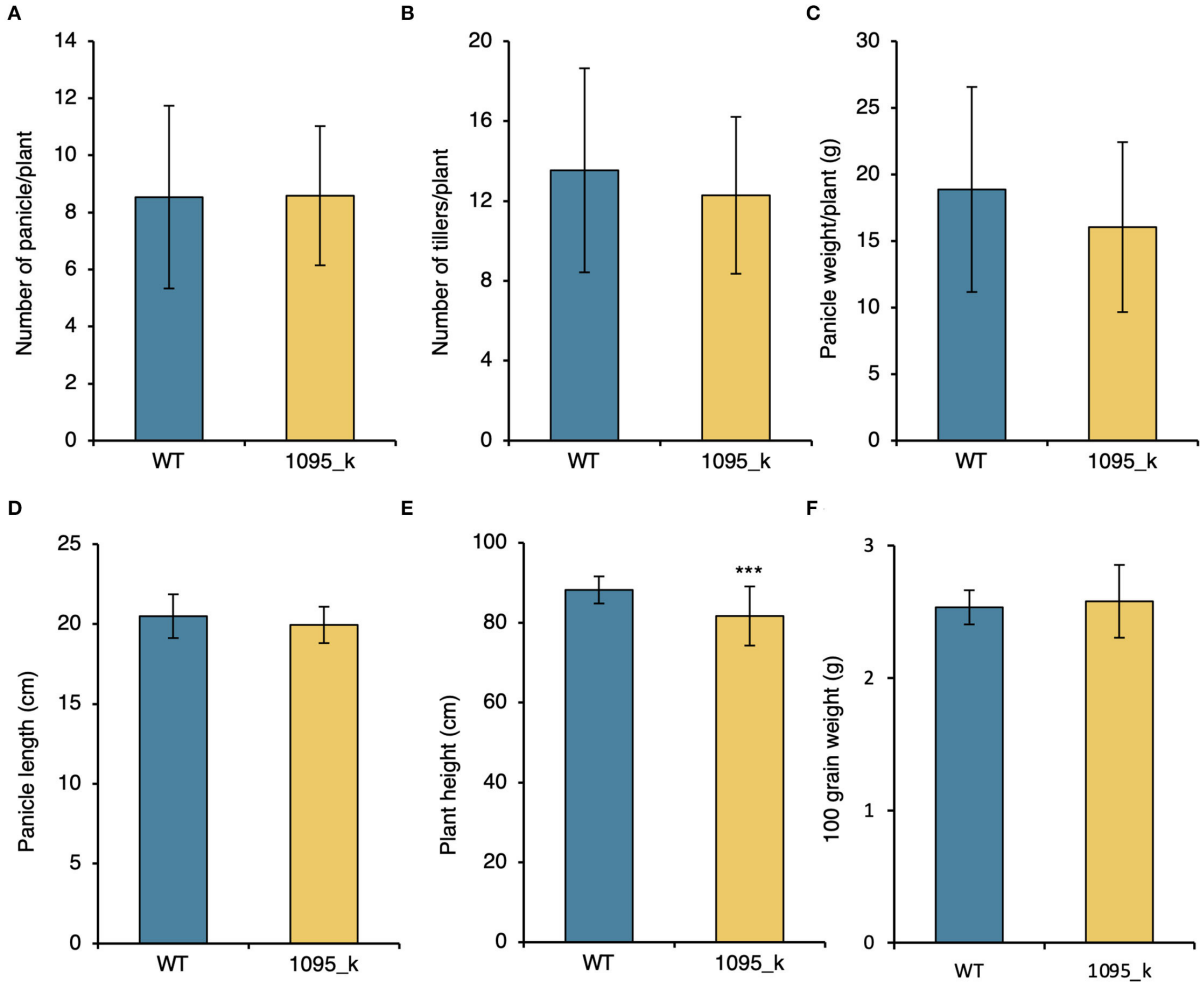
Characteristics of the high Fe mutant. **(A)** Number of panicle/plant, **(B)** number of tillers/plant, **(C)** panicle weight/plant, **(D)** Panicle length, **(E)** Plant height, and **(F)** 100 grain weight of WT and 1095_k. Plants were harvested from the paddy field after maturation. Significant difference using Student's *t*-test. *** *p* < 0.001. Error bar = SD. *n* = 41.

Agronomic characters are an essential factor when considering breeding. Both WT and mutant 1095_k were cultivated in the Tokyo field. In each line, 41 plants were cultivated. The following yield components were measured: number of panicles/plant, number of tillers/plant, panicle weight/plant, panicle length, plant height, and 100 grain weight. No significant difference was observed in yield components between WT and 1095_k (Figures 2A–D,F, Figure S2B). The plant height was significantly smaller in 1095_k than that of the WT (Figure 2E, Figure S2A). These results suggest that 1095_k is a potential mutant that can retain high Fe and Zn without compromising yield components.

### *OsVIT2* Is a Possible Candidate for the Causal Gene of 1095_k

**Figure 3:**
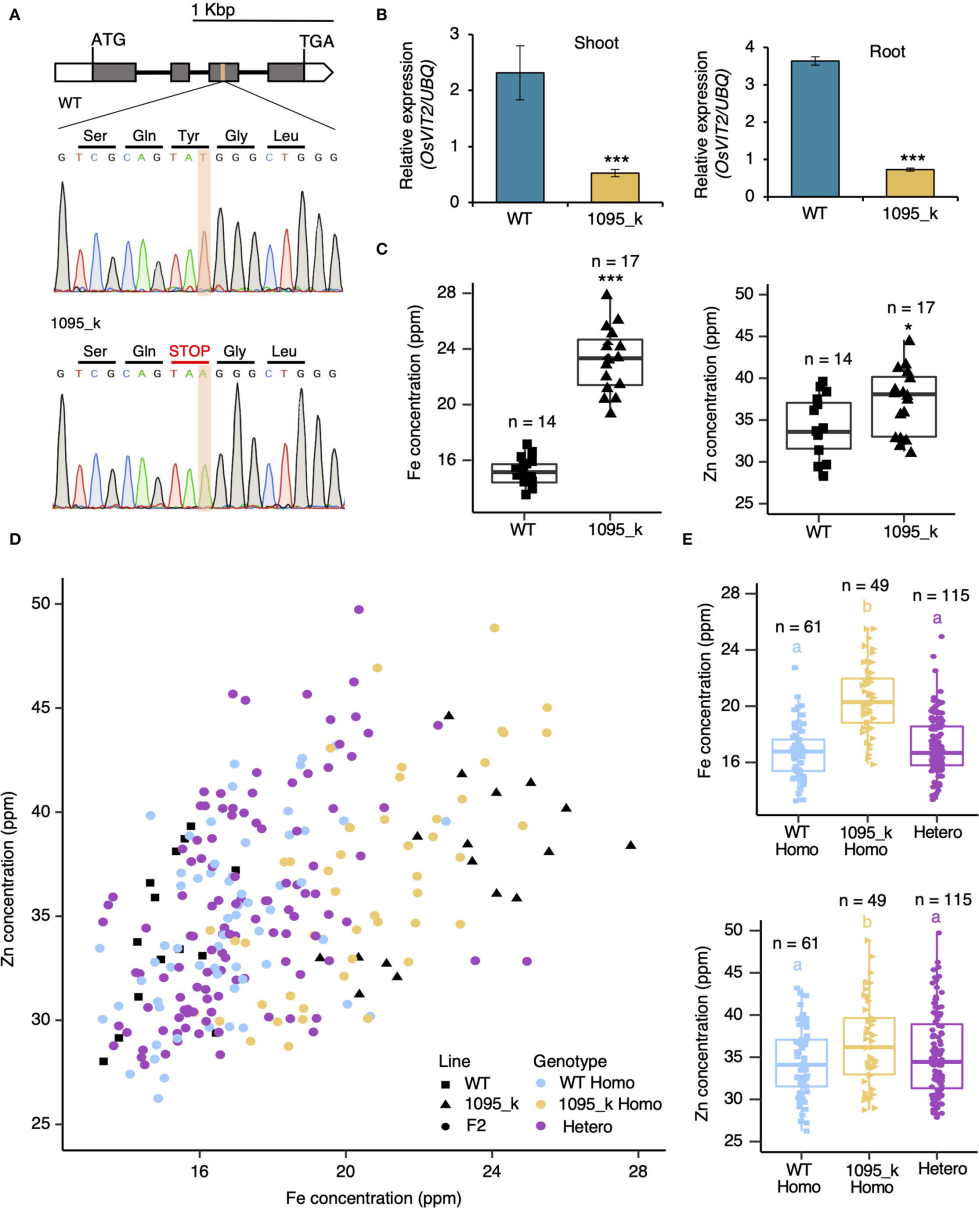
The correlation between mutated OsVIT2 and high Fe phenotype. **(A)** Intron-exon structure of *OsVIT2* with sequence alignment of WT and 1095_k (box and line are exon and intron, whereas white box and gray box represent UTR and CDS, respectively). Nucleotide changes are indicated by the red box in the sequence spectrum. Thymine in WT is changed to Adenine in 1095_k at the third exon of *OsVIT2*. **(B)** The mRNA expression of *OsVIT2* relative to the *UBQ* gene in shoot and root samples from WT and 1095_k. Plants were grown for 1 week in normal Kimura's B solution. Significant differences were calculated using Student's *t*-test. *** *p* < 0.001. Error bar = SD. *n* = 3. **(C)** Fe and Zn concentration of WT and 1095_k in brown rice. Plants were cultivated in the rice field of the Yayoi campus, The University of Tokyo. Significant differences were calculated using Student's *t*-test. * *p* < 0.05, *** *p* < 0.001. Error bar = SD. **(D)** A correlation plot between Fe and Zn concentration in brown rice for each genotype in the F2 population. F2 plants were grown together with parental line **(C)** and Inductive Coupled Plasma Mass Spectrometry (ICP-MS) analysis was performed. WT and 1095_k are the same data as in **(C, E)** Boxplot of different F2 genotypes in **(C)**. Significant differences were calculated using Tukey's HSD (*p* < 0.05). Letters indicate significant differences. The number of replicates is shown in figure.

Several genes have been identified to be involved in the transport of Fe and Zn to the grain of rice. Fe transporters, *OsVIT1* and *OsVIT2*, have been characterized, whose T-DNA insertion lines show the increase in Fe and Zn concentration of grain (Zhang et al., 2012; Bashir et al., 2013). In 1095_k, there is an increase in Fe and Zn concentration in grains similarly to the mutant of *OsVIT*s (Figure 1). Therefore, we hypothesized that the alteration in *OsVIT* genes might be a cause of the phenotype. To test this, the genomic sequence of *OsVIT1* and *OsVIT2* was determined: the promoter (2,078 bp for *OsVIT1* and 2,035 bp for *OsVIT2*), gene body, and downstream sequence (1,002 bp of *OsVIT1* and 2,047 bp for *OsVIT2*). There is no nucleotide change in the *OsVIT1*. In *OsVIT2*, there is a nonsense mutation in the third exon, where thymine (T) at the 360th position in CDS was replaced by adenine (A), resulting in the formation of the stop codon (TAA) (Figure 3A). The relative mRNA accumulation of *OsVIT2* is reduced in 1095_k both in shoot and roots (Figure 3B), which might be due to nonsense-mediated mRNA decay. These data suggest that nonsense mutation in *OsVIT2* of 1095_k is a possible candidate gene behind the high Fe and Zn phenotype.

To confirm if *OsVIT2* is a causal gene of 1095_k, we observed the correlation between genotype and element concentrations in the F2 crosses between WT and 1095_k. The plants were grown in the Yayoi field, and the concentrations of the elements were determined by ICP-MS. The Fe and Zn concentration increase was observed in 1095_k compared with WT in this field, which confirms that the Yayoi field condition can be used for the F2 population phenotyping (Figure 3C). Fe and Zn concentrations were determined in F2 populations and there is a positive correlation (Pearson's *r* = 0.418) between Fe and Zn concentration (Figure 3D), suggesting that high Fe and Zn phenotype is caused by the same gene. Next, we observed the relationship between the *OsVIT2* genotype and Fe or Zn concentration in the F2 population. The genotype of all the F2 (225 lines) was determined by the dCAPS marker (Figure 3E). To see the association between genotype and phenotype, a statistical analysis was performed for Fe and Zn concentration (Figure 3D). For both Fe and Zn concentrations, there is a statistical significance between the wild-type homozygous and mutant-type homozygous lines (WT Homo and 1095_k Homo in Figures 3D,E). These results strongly suggest that nonsense mutation of *OsVIT2* is responsible for the high Fe and Zn phenotypes in 1095_k.

## Discussion

### *OsVIT2* Could Be a Possible Causal Gene for 1095_k

An EMS mutant, 1095_k, has an increase in Fe and Zn concentration in brown rice as well as in polished rice (Figures 1, 3C). Furthermore, there is an association between Fe and Zn concentration and *OsVIT2* mutation in F2 crosses between WT and 1095_k. The OsVIT2 is a vacuolar localized transporter, which plays an important role in the vacuolar sequestration of Fe to regulate Fe homeostasis (Zhang et al., 2012; Bashir et al., 2013). It has been demonstrated that the disruption of *OsVIT2* results in an increase in Fe and Zn concentration in rice grains. In addition to these elements, Cu and Mn change, although it varies from experiment to experiment: Cu increases in *OsVIT2* mutant but no change in Mn (Bashir et al., 2013); no change both in Cu and Mn (Zhang et al., 2012). In terms of plant growth, the *OsVIT2* mutant shows a decrease in shoot length at the seedling stage (Zhang et al., 2012). Although we did not perform the growth test in the seedling stage, in the field test, the plant height of 1095_k was lower than WT (Figure 2E). Taken together, these results suggest that *OsVIT2* could be a causal gene for the high grain Fe and Zn phenotype of 1095_k.

### 1095_k Can Be a Material for Breeding High Fe and Zn Cultivars

In 1095_k, *OsVIT2* mutation increased Fe and Zn content of grain in fields without increasing toxic elements, such as Cd and As, for humans (Figure 1 and Figure S1). In addition, we showed that the mutation does not affect the growth of rice, and furthermore, no reduction in the yield components was observed (Figure 2 and Figure S2). These results indicate that the mutation in *OsVIT2* could be a candidate for DNA marker to breed high Fe and Zn. All of these characters are beneficial for breeding new cultivars.

OsVIT2 is localized to vacuole and sequester Fe into vacuole (Zhang et al., 2012). *OsVIT2* mRNA is expressed throughout whole growth stages (Bashir et al., 2013) and upregulated by high Fe in the seedling stage (Zhang et al., 2012). These results suggest the function of OsVIT2 in excess Fe tolerance. Bashir et al. (2013) grew the T-DNA knockdown line under excess Fe conditions and found that under 100 and 500 μM Fe-EDTA conditions, the plant height is shorter than that of control plants, while root length and soil plant analysis development (SPAD) values are similar between them. Therefore, OsVIT2 may have some function in excess Fe tolerance but may not affect the growth severely. In our experiments, although the plant height is short at 1095_k, the yield of 1095_k is similar to WT. Therefore, the yield of the *OsVIT2* mutant would not be affected so much. The other possible disadvantage is the high accumulation of toxic elements in humans, such as Cd and As. In our field condition and also in Cd-contaminated soil (Che et al., 2021), there is no significant difference between WT and mutants. Taken together with yield results, *OsVIT2* is suitable as a breeding material.

There have been many efforts made in the past to increase the Fe and Zn concentration in the rice grains, but are generated by the transgenic means due to which they are restricted to use around the world (Takahashi et al., 2001; Lee and An, 2009; Zheng et al., 2010). In the case of 1095_k, an EMS mutant, the freedom of usage increases compared with genetically modified rice. Furthermore, if transgenic lines are accepted, breeders can use 1095_k as a material for transformation by overexpression of genes enhancing Fe and/or Zn concentration in grain as the mutant does not have any selection marker (e.g., hygromycin) gene. Hence, mutant 1095_k is a strong candidate to address the hidden hunger problem in the coming future.

## Data Availability Statement

The original contributions presented in the study are included in the article/Supplementary Material, further inquiries can be directed to the corresponding author/s.

## Author Contributions

PK, TF, and TK conceived and designed the experiment. PK and TK performed the experiments, and data analysis and, wrote the manuscript. All authors contributed to the article and approved the submitted version.

## Funding

This work was supported by JST, PRESTO grant number JPMJPR16Q3, Science and Technology Research Partnership for Sustainable Development (SATREPS), Japan Science and Technology Agency (JST)/Japan International Cooperation Agency (JICA) grant number JPMJSA2107, and Steel Foundation for Environmental Protection Technology to TK. It is also supported in part by the JSPS KAKENHI 18H05490 and 19H05637, Cabinet Office, Government of Japan, Moonshot R&D Program for Agriculture, Forestry, and Fisheries (founding agency: 252 Bio-oriented Technology Research Advancement Institution) to TF.

## Conflict of Interest

The authors declare that the research was conducted in the absence of any commercial or financial relationships that could be construed as a potential conflict of interest.

## Publisher's Note

All claims expressed in this article are solely those of the authors and do not necessarily represent those of their affiliated organizations, or those of the publisher, the editors and the reviewers. Any product that may be evaluated in this article, or claim that may be made by its manufacturer, is not guaranteed or endorsed by the publisher.
