# Supplementary material for: OsVIT2 Mutation Increases Fe and Zn of Grain Without Compromising the Growth in Paddy Field
Source: Front Plant Sci. 2022 Jun 22;13:868661. doi: 10.3389/fpls.2022.868661 (PMC9257253; doi:10.3389/fpls.2022.868661)
Supplement: Supplementary file 1 [file Presentation_1.PPTX]

## Slide 1
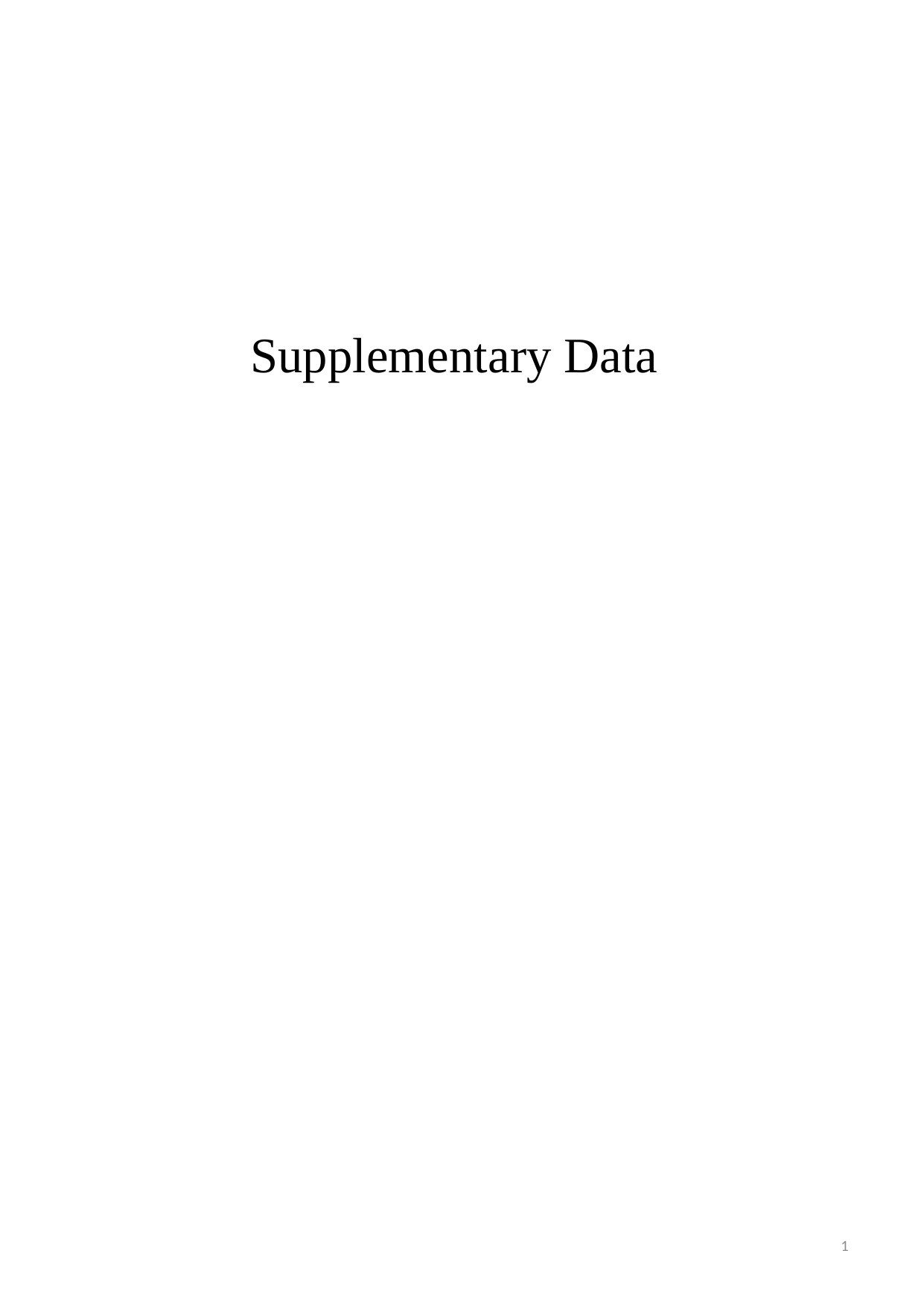

Supplementary Data
1

## Slide 2
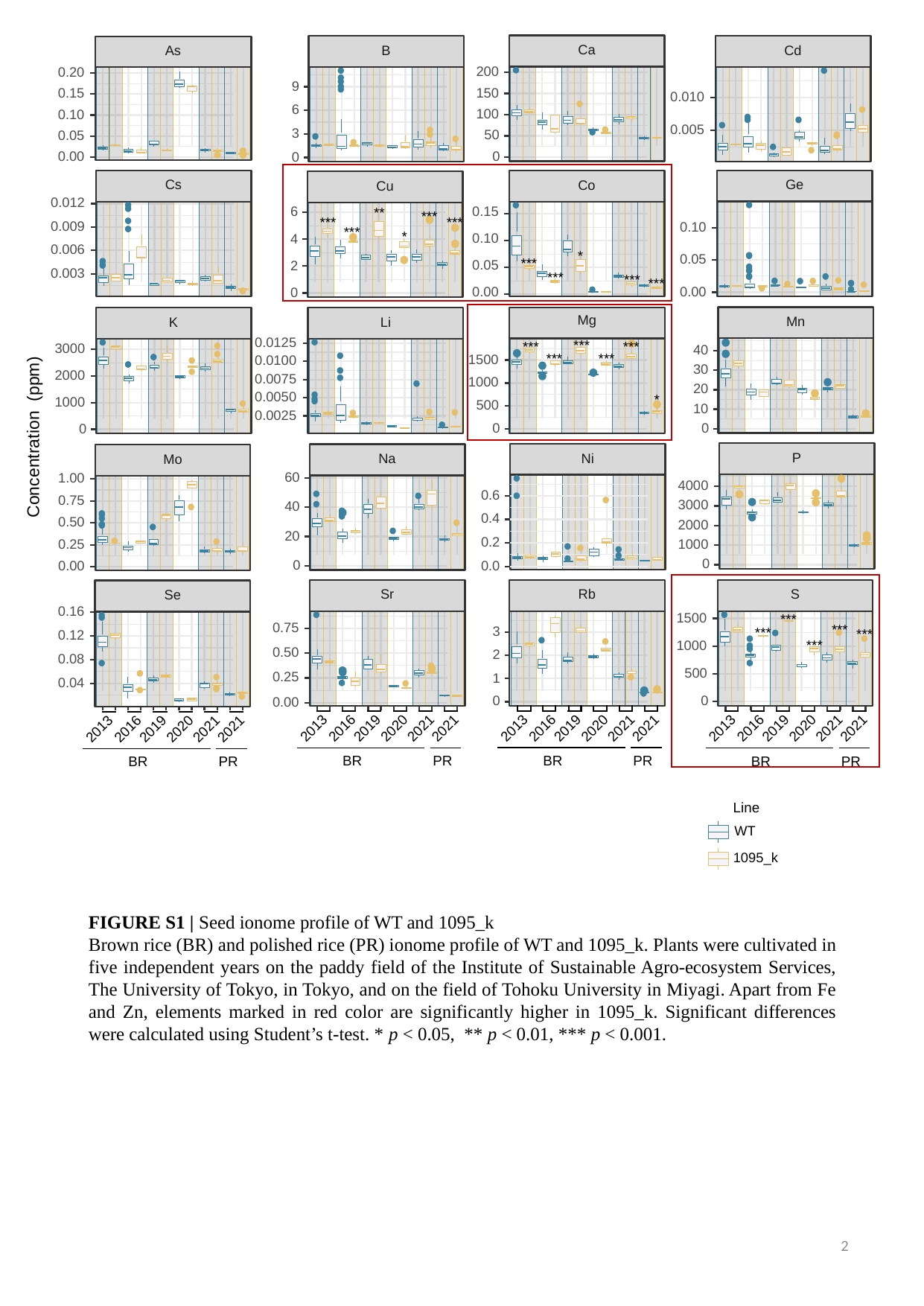

Ca
200
150
100
50
0
Cd
0.010
0.005
B
9
6
3
0
As
0.20
0.15
0.10
0.05
0.00
Ge
0.10
0.05
0.00
Cs
0.012
0.009
0.006
0.003
Co
0.15
0.10
0.05
0.00
*
***
***
***
***
Cu
6
4
2
0
**
***
***
***
***
*
Mn
40
30
20
10
0
Mg
500
0
1500
1000
***
***
***
***
***
*
Li
0.0125
0.0100
0.0075
0.0050
0.0025
K
3000
2000
1000
0
Concentration (ppm)
P
4000
3000
2000
1000
0
Ni
0.6
0.4
0.2
0.0
Na
60
40
20
0
Mo
1.00
0.75
0.50
0.25
0.00
Rb
3
2
1
0
S
1500
1000
500
0
***
***
***
***
***
Sr
0.75
0.50
0.25
0.00
Se
0.16
0.12
0.08
0.04
2013
2016
2019
2020
2021
2021
BR
PR
2013
2016
2019
2020
2021
2021
BR
PR
2013
2016
2019
2020
2021
2021
BR
PR
2013
2016
2019
2020
2021
2021
BR
PR
Line
WT
1095_k
FIGURE S1 | Seed ionome profile of WT and 1095_k
Brown rice (BR) and polished rice (PR) ionome profile of WT and 1095_k. Plants were cultivated in five independent years on the paddy field of the Institute of Sustainable Agro-ecosystem Services, The University of Tokyo, in Tokyo, and on the field of Tohoku University in Miyagi. Apart from Fe and Zn, elements marked in red color are significantly higher in 1095_k. Significant differences were calculated using Student’s t-test. * p < 0.05, ** p < 0.01, *** p < 0.001.
2

## Slide 3
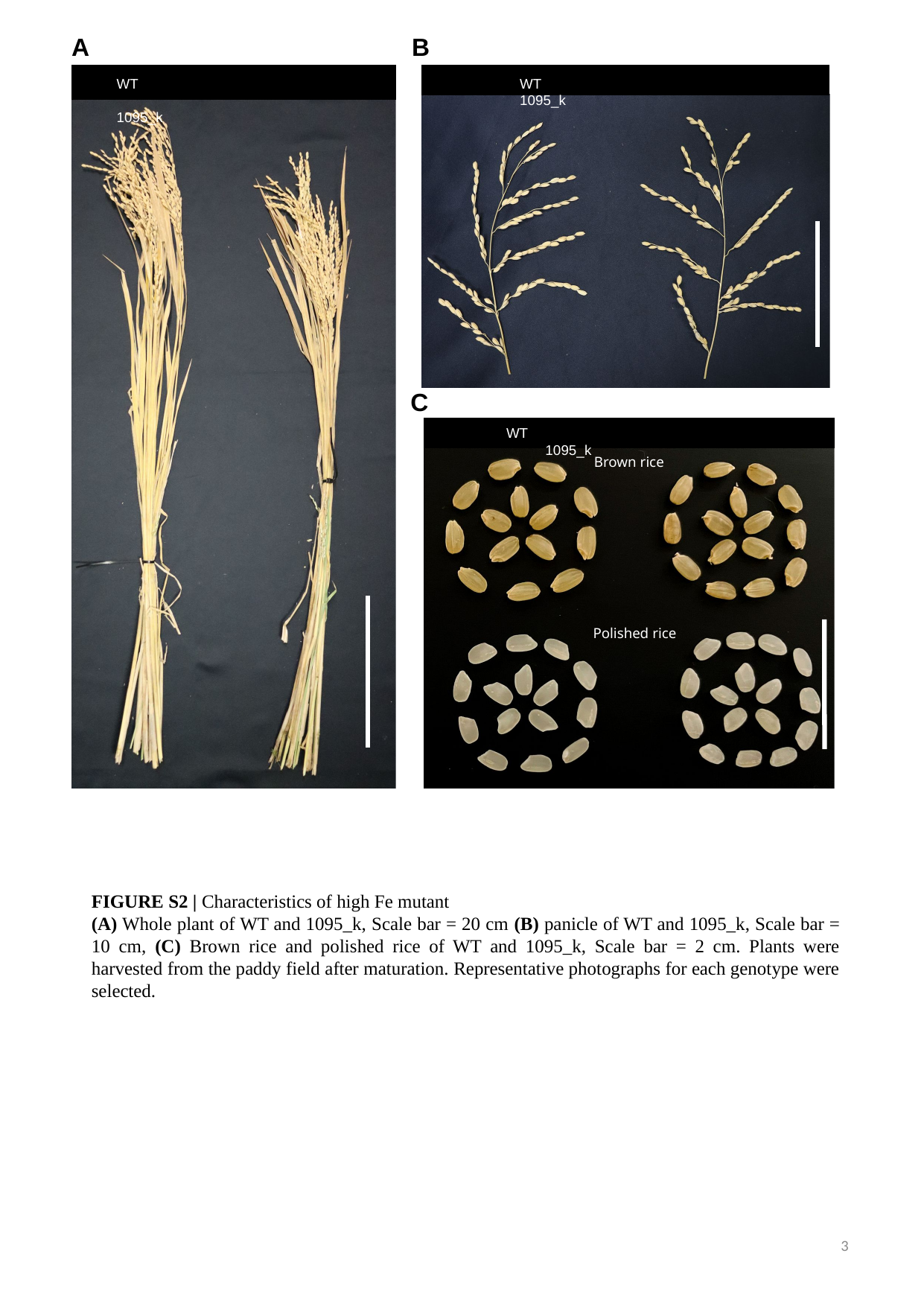

A
B
WT		 1095_k
WT 		 1095_k
C
WT 		 1095_k
Brown rice
Polished rice
FIGURE S2 | Characteristics of high Fe mutant
(A) Whole plant of WT and 1095_k, Scale bar = 20 cm (B) panicle of WT and 1095_k, Scale bar = 10 cm, (C) Brown rice and polished rice of WT and 1095_k, Scale bar = 2 cm. Plants were harvested from the paddy field after maturation. Representative photographs for each genotype were selected.
3
